# Associations of childhood, adolescence, and midlife cognitive function with DNA methylation age acceleration in midlife

**DOI:** 10.18632/aging.205943

**Published:** 2024-06-13

**Authors:** Junyu Chen, Leah Moubadder, Elizabeth S. Clausing, Katrina L. Kezios, Karen N. Conneely, Anke Hüls, Andrea Baccarelli, Pam Factor-Litvak, Piera Cirrillo, Rachel C. Shelton, Bruce G. Link, Shakira F. Suglia

**Affiliations:** 1 Department of Epidemiology, Rollins School of Public Health, Emory University, Atlanta, GA 30322, USA; 2 School of Global Integrative Studies, University of Nebraska, Lincoln, NE 68508, USA; 3 Department of Epidemiology, Mailman School of Public Health, Columbia University, New York, NY 10032, USA; 4 Department of Human Genetics, School of Medicine, Emory University, Atlanta, GA 30322, USA; 5 Gangarosa Department of Environmental Health, Rollins School of Public Health, Emory University, Atlanta, GA 30322, USA; 6 Department of Environmental Health Sciences, Mailman School of Public Health, Columbia University, New York, NY 10032, USA; 7 Child Health and Development Studies, Public Health Institute, Washington, DC 20024, USA; 8 Department of Sociomedical Sciences, Mailman School of Public Health, Columbia University, New York, NY 10032, USA; 9 Department of Sociology, University of California Riverside, Riverside, CA 92507, USA

**Keywords:** age acceleration, adolescent cognition, adult cognition, biological aging, epigenetics

## Abstract

Prior studies showed increased age acceleration (AgeAccel) is associated with worse cognitive function among old adults. We examine the associations of childhood, adolescence and midlife cognition with AgeAccel based on DNA methylation (DNAm) in midlife.

Data are from 359 participants who had cognition measured in childhood and adolescence in the Child Health and Development study, and had cognition, blood based DNAm measured during midlife in the Disparities study. Childhood cognition was measured by Raven’s Progressive Matrices and Peabody Picture Vocabulary Test (PPVT). Adolescent cognition was measured only by PPVT. Midlife cognition included Wechsler Test of Adult Reading (WTAR), Verbal Fluency (VF), Digit Symbol (DS). AgeAccel measures including Horvath, Hannum, PhenoAge, GrimAge and DunedinPACE were calculated from DNAm. Linear regressions adjusted for potential confounders were utilized to examine the association between each cognitive measure in relation to each AgeAccel.

There are no significant associations between childhood cognition and midlife AgeAccel. A 1-unit increase in adolescent PPVT, which measures crystalized intelligence, is associated with 0.048-year decrease of aging measured by GrimAge and this association is attenuated after adjustment for adult socioeconomic status. Midlife crystalized intelligence measure WTAR is negatively associated with PhenoAge and DunedinPACE, and midlife fluid intelligence measure (DS) is negatively associated with GrimAge, PhenoAge and DunedinPACE. AgeAccel is not associated with VF in midlife.

In conclusion, our study showed the potential role of cognitive functions at younger ages in the process of biological aging. We also showed a potential relationship of both crystalized and fluid intelligence with aging acceleration.

## INTRODUCTION

Many neurodegenerative pathologies such as amyloid plaques and neurofibrillary tangles develop long before the clinical diagnosis of cognitive disorders [1]. This has led to many investigations of early-life cognitive outcomes, which have been demonstrated to be instructive in studying late-life cognitive impairment and dementia [2]. Recent research showed that both level of and change in language-based cognition during adolescence were associated with midlife cognition [3], suggesting that prevention of cognitive disorders may be able to start as early as adolescence.

DNA methylation (DNAm), which can regulate gene expression without altering the DNA segments by adding a methyl group at cytosine residues, has been found to be associated with cognitive abilities [4]. DNAm may contribute to learning and memory through a variety of mechanisms including synaptic plasticity and neurogenesis, stem cell function, immunosenescence, circadian rhythms, and the effects of cumulative stress through glucocorticoid signaling [5]. In recent years, epigenetic clocks were created based on DNAm to estimate the construct of ‘biological’ age [6–10]. DNAm age accelerations, defined as the difference between epigenetic clocks and chronological age or the residuals in epigenetic clocks independent of chronological age, were proposed to measure the discrepancy between chronological and biological age. These DNAm age accelerations have been repeatedly shown to be associated with early-life exposures and diverse age-related disorders and premature mortality [11–15]. Investigating the relationship between cognitive functions and DNAm age acceleration could be useful for identification of mechanisms underlying the development of cognitive disorder independent of known neuro-pathologies.

To date, only a handful of studies have examined the relationship between cognitive function and DNAm age acceleration. While previous evidence from a small number of cross-sectional studies was inconsistent [5, 16–19], three recent longitudinal studies on cognition at older age (>65 years old) have found that a faster age acceleration was associated with cognitive decline [5, 20, 21]. Two other studies showed that the negative associations between DNAm age acceleration and cognitive skills may exist in late adolescence [22, 23]. However, the relationship between cognitive function and DNAm age acceleration from adolescence to midlife hasn’t been explored. In the current study, we investigated whether cognitive function in childhood and adolescence is related to DNAm age acceleration assessed 35 years after the cognitive function assessment. Many studies have demonstrated the role of epigenetic modifications in cognitive aging at old age [5, 20, 21, 24]. Others have suggested DNAm age acceleration is a mediator for sex differences in verbal memory and processing speed [25, 26]. Thus, we hypothesized that the DNAm age acceleration influenced by cognitive skills at early age could in turn affect one’s cognition in later life and examined the cross-sectional associations of DNAm age acceleration and cognitive measures in midlife. By using this unique study design, we hope to make the first step to understand the relationship between cognition at younger age and aging, which might potentially help refine the strategy to prevent cognitive aging in the future.

## RESULTS

**Figure 1:**
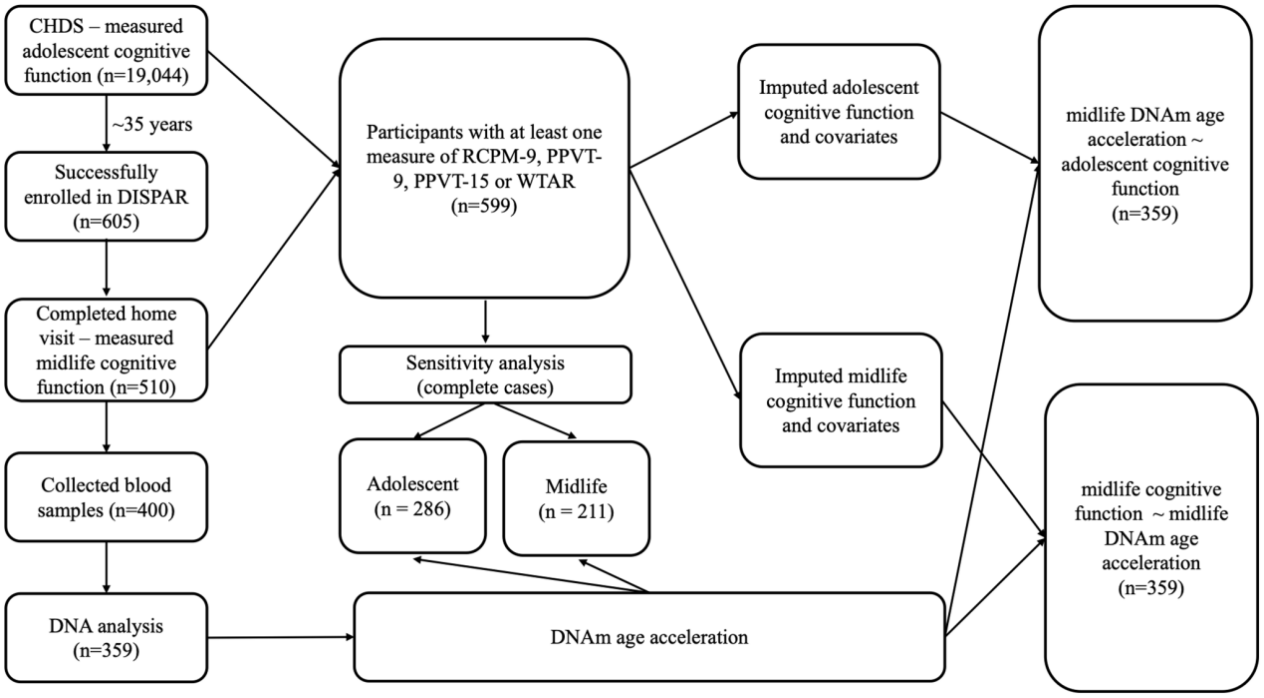
Overview of study population and workflow.

We provide an overview of the study population and workflow in Figure 1. Briefly, data from 359 participants who had cognition measured in childhood and adolescence in the Child Health and Development study (CHDS), and had cognition, blood based DNAm measured during midlife in the Disparities study (DISPAR) were used. Childhood (9–11 years) fluid intelligence was measured by Raven Colored Progressive Matrices (RCPM-9) and crystallized intelligence was measured by Peabody Picture Vocabulary Test (PPVT-9). Adolescent (15–17) cognition was measured only by Peabody Picture Vocabulary Test (PPVT-15). Midlife crystallized intelligence was measured by Wechsler Test of Adult Reading (WTAR) and fluid intelligence was measured by Verbal Fluency (VF), Digit Symbol (DS). Midlife DNAm age acceleration measures including Horvath, Hannum, PhenoAge, GrimAge and DunedinPACE were calculated from DNAm. Linear regressions adjusted for potential confounders were utilized to examine the associations between each cognitive measure in relation to each DNAm age acceleration. To align with our hypotheses mentioned above, childhood/adolescent cognitive measures were used as independent variables in the childhood/adolescent analyses while midlife cognitive measures were used as dependent variables in the midlife analyses.

### Characteristics of analytic sample

Sociodemographic and clinical characteristics of the participants are summarized in Table 1. Approximately half of the study population are female (46.8%). Over half are non-Hispanic White (56.5%) and 61 (34.3%) are non-Hispanic Black based on self-identified race of the participants in the DISPAR study. Participants who did not self-identify as non-Hispanic White or Black are included as “Other race/ethnicity” (*n* = 33).

**Table 1:** Demographic and clinical characteristics of the child health and development studies (CHDS) and the racial disparities (DISPAR) Aging study participants in childhood, adolescence, and midlife (*n* = 359).

	**Childhood**	**Adolescence**	**Midlife**
**Female**, *n* (%)	168 (46.8%)		
**Race/Ethnicity**, *n* (%)			
** Non-Hispanic White**	203 (56.5%)		
** Non-Hispanic Black**	123 (34.3%)		
** Other Race/Ethnicity**	33 (9.2%)		
**Age**, mean (SD)	10.0 (0.9)	16.6 (0.6)	49.3 (1.3)
**RCPM**, mean (SD)	32.3 (10.2)	–	–
**PPVT**, mean (SD)	81.3 (11.2)	114 (16.5)	–
**WTAR**, mean (SD)	–	–	33.8 (10.2)
**VF**, mean (SD)	–	–	23.4 (6.1)
**DS**, mean (SD)	–	–	58.8 (18.1)
**Socioeconomic status^1^**, *n* (%)			
** Low**	119 (33.1%)	119 (33.0%)	
** Medium**	178 (49.6%)	155 (43.2%)	
** High**	62 (17.3%)	85 (23.7%)	
**BMI**, mean (SD)	17.7 (2.7)	22.0 (4.10)	29.6 (6.4)
**Lived with someone that smoked**, *n* (%)	215 (59.9%)	215 (59.9%)	–
**Cigarette use^2^**, *n* (%)			
** Never**	–	307 (85.5%)	192 (53.5%)
** Past**	–	46 (12.8%)	90 (25.1%)
** Current**	–	6 (1.7%)	77 (21.4%)
**Alcohol use^2^**, *n* (%)			
** Never**	–	86 (24.0%)	83 (23.1%)
** Past**	–	–	77 (21.4%)
** Current**	–	273 (76.0%)	199 (55.4%)
**Hypertension**, *n* (%)	–	–	98 (27.3%)
**Horvath^3^**, mean (SD)	–	–	0.0 (4.34)
**Hannum^3^**, mean (SD)	–	–	0.0 (4.11)
**GrimAge^3^**, mean (SD)	–	–	0.0 (4.39)
**PhenoAge^3^**, mean (SD)	–	–	0.0 (5.94)
**DunedinPACE**, mean (SD)	–	–	1.0 (0.136)

Abbreviation: RCPM: Raven Colored Progressive Matrices; PPVT: Peabody Picture Vocabulary Test; WTAR: Wechsler Test of Adult Reading; VF: Verbal Fluency; DS: Digit Symbol; SES: Socioeconomic Status; BMI: Body Mass Index. ^1^One SES measure was created based on maternal education at birth, paternal occupation, and family income at birth, ages 9–11 and 15–17 to represent SES at both childhood and adolescence. Midlife SES index was developed by combining information on college education, self-reported family income and occupational status. ^2^Information on cigarette and alcohol use in adolescence were measured and defined in difference matrices from those in midlife. ^3^Horvath, Hannum, GrimAge and PhenoAge are the residuals in the corresponding epigenetic clocks after regressing out chronological age.

All 5 DNAm age acceleration measures follow a normal distribution (Supplementary Figure 1) but DunedinPACE has a much smaller standard deviation (SD) (Table 1). We show that DunedinPACE has a moderate correlation with GrimAge (R = 0.6) but neither measure correlates with Horvath or Hannum (R ≤ 0.15) (Supplementary Figure 2C). PhenoAge has a weak positive correlation with all other age acceleration measures (R ≈ 0.4).

All cognitive scores were comparable to average scores in the general population. All cognitive measures in childhood and adolescence are positively correlated with each other before and after multiple imputation (Supplementary Figure 2A, 2B). Distributions of midlife cognitive function are similar before and after multiple imputation (Supplementary Figure 3). VF and DS follow a normal distribution and WTAR is moderately left-skewed. WTAR shows moderate correlations with all childhood and adolescent cognitive measures, especially PPVT-15 (R = 0.77) as they all measure crystallized intelligence that’s developed at an early age (Supplementary Figure 2B). The three midlife cognitive measures have low correlations with each other (R ranges 0.21–0.4), given they assess different components of adult cognition [27–30].

### Childhood/adolescent cognitive function and midlife DNAm age acceleration

**Figure 2:**
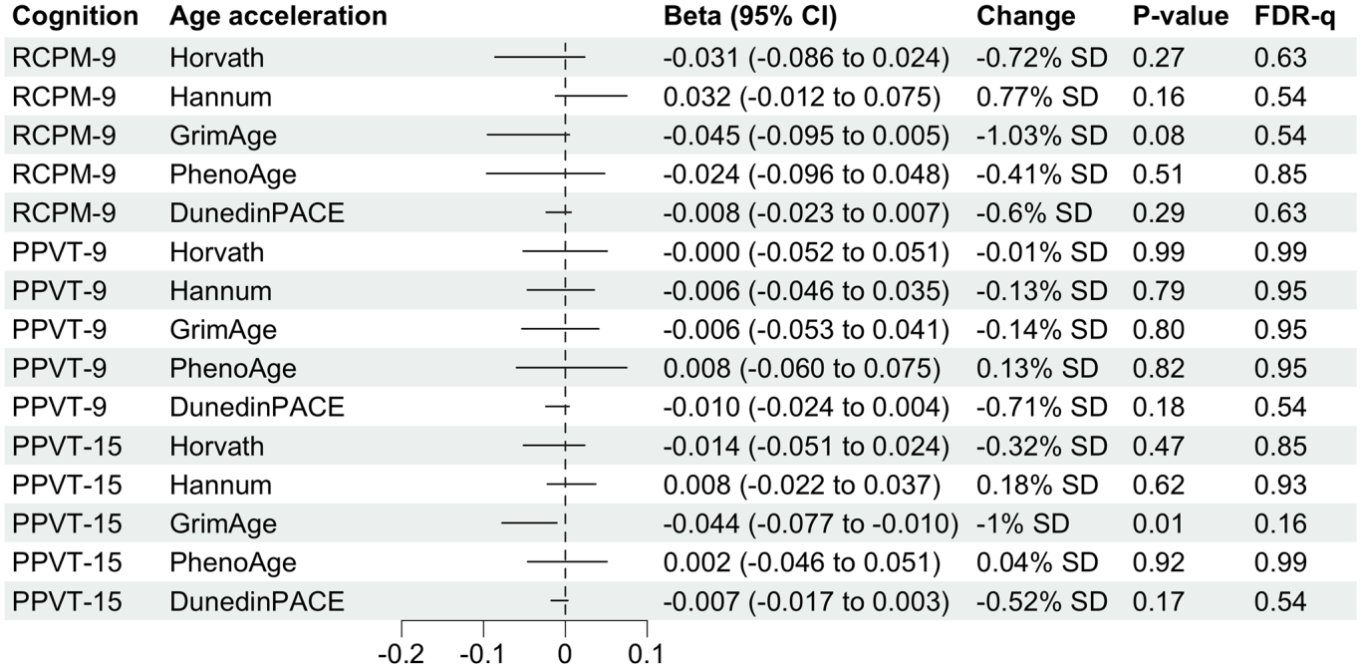
**Forest plot showing associations of childhood and adolescent cognitive function with midlife DNA methylation age accelerations.** Betas multiplied by 10 were shown for associations with DunedinPACE for better visualization. Column “Change” is showing that 1-unit change in childhood or adolescent cognitive function is associated with X.XX% standard deviation (SD) change in DNA methylation age acceleration. SD for each DNA methylation age can be found in Table 1.

There are no statistically significant associations between childhood cognitive function and midlife DNAm age acceleration measures (Figure 2). Adolescent cognitive measure (PPVT-15) is not significantly associated with Horvath, Hannam and PhenoAge age acceleration but GrimAge and DunedinPACE. A 1-unit increase in PPVT-15 is associated with 0.048-year (1.1% SD) decrease of aging measured by GrimAge and 0.001 decrease (0.71% SD) in the pace of aging measured by DunedinPACE (Figure 2). However, the latter is not statistically significant after adjusting for multiple testing. Associations from complete case analyses are similar to the ones from analyses after multiple imputation (Supplementary Figure 4). After adjusting for adulthood SES, the absolute value of Beta for the associations of PPVT-15 with GrimAge and DunedinPACE has shrunk to half (Table 2).

**Table 2:** Comparison of associations of adolescent cognitive function (PPVT-15) with midlife DNA methylation age accelerations before and after adjustment for adult SES.

	**Before adjustment for adult SES**				**After adjustment for adult SES**			
**Age acceleration**	**Beta**	**SE**	**Age acceleration**	**Beta**	**SE**	**Age acceleration**	**Beta**	**SE**
Horvath	−0.018	0.016	0.283	−0.406%	−0.014	0.017	0.413	−0.323%
Hannum	0.012	0.013	0.373	0.285%	0.011	0.014	0.421	0.268%
GrimAge	−0.048	0.015	1.693E-03	−1.101%	−0.025	0.015	0.105	−0.566%
PhenoAge	−0.009	0.021	0.665	−0.154%	−0.005	0.022	0.804	−0.092%
DunedinPACE	−9.653E-03	4.453E-03	0.031	−0.710%	−4.521E-03	4.549E-03	0.321	−0.332%

Column “% SD change” is showing that 1-unit change in PPVT-15 is associated with X.XXX% standard deviation (SD) change in DNA methylation age acceleration. SD for each DNA methylation age can be found in Table 1.

### Midlife cognitive function and midlife DNAm age acceleration

**Figure 3:**
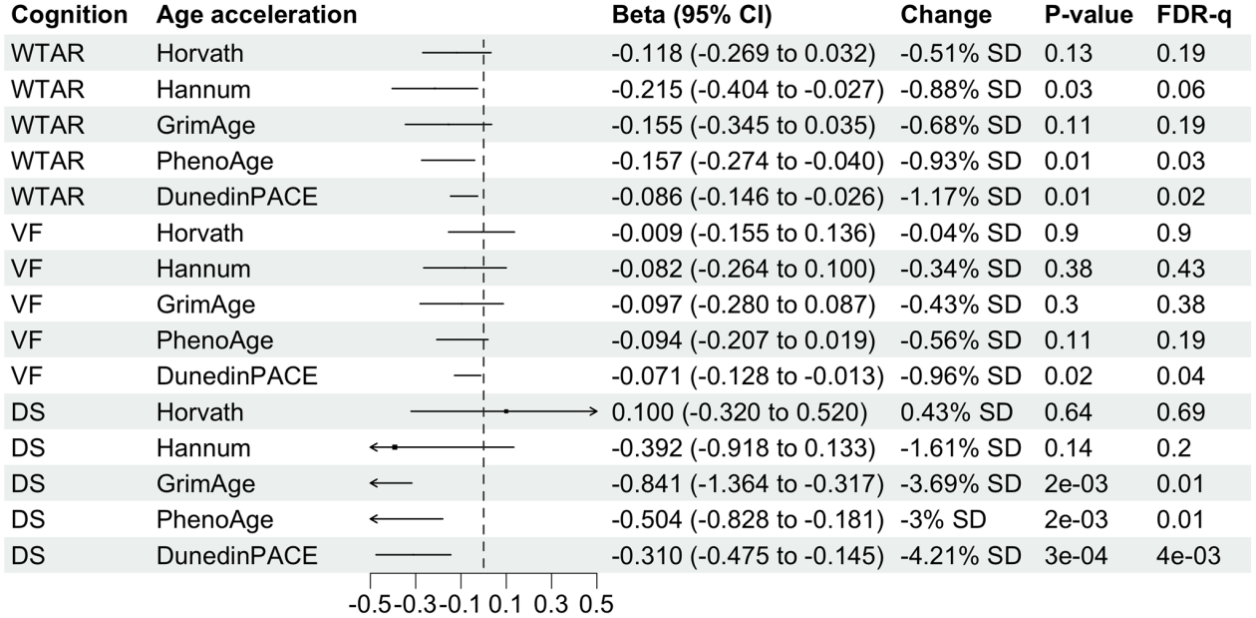
**Forest plot showing associations of midlife DNA methylation age acceleration with midlife cognitive function.** Betas divided by 100 were shown for associations with DunedinPACE for better visualization. Column “Change” is showing that 1-standard deviation (SD) change in DNA methylation age acceleration is associated with X.XX-unit change in midlife cognitive function. SD for each DNA methylation age can be found in Table 1.

WTAR shows negative associations with all 5 age acceleration measures but only two are statistically significant with FDR-q value less than 0.05 (Figure 3). Specifically, 1-SD increase in PhenoAge is associated with 0.89-unit decrease in WTAR score. A 1-SD increase in DunedinPACE is associated with 1.12-unit decrease in DunedinPACE. Associations between WTAR on PhenoAge and DunedinPACE are no longer in complete case analysis than analysis after multiple imputation (Supplementary Figure 5).

VF test score decreases by 0.94 per SD increase of pace of aging measured by DunedinPACE and remains significant after adjusting for multiple testing (Figure 3). Although the estimated beta coefficient is similar to the one in the complete case analysis, it is no longer significant in the complete case analysis (Supplementary Figure 5). VF is not significantly associated with other age acceleration measures.

DS is not significantly associated with Horvath and Hannum age acceleration (Figure 3). A 1-SD year increase in aging measured by GrimAge, PhenoAge and DunedinPACE is associated with 3.57, 2.91 and 4.1 decrease in DS respectively. These three associations remain significant even after multiple testing adjustment, however, the precision of the estimates is low (wide 95% confidence interval). In complete case analysis, associations are larger than analysis after multiple imputation, with 1-SD year increase in GrimAge, PhenoAge or DunedinPACE associated with more than 4-unit decrease in DS.

## DISCUSSION

We investigated the associations between childhood, adolescent and midlife cognitive function with DNAm age acceleration in midlife. Our findings show that those with poorer crystalized intelligence (PPVT-15) in adolescence are more likely to have accelerated GrimAge in their midlife. We also show that accelerated PhenoAge and DunedinPACE in midlife is associated with both reduced crystalized intelligence (WTAR) and fluid intelligence (DS), while accelerated GrimAge in midlife is only associated with crystalized intelligence (WTAR). In the complete case analyses, the magnitude of the associations is similar, however, only the associations of DNAm age acceleration with fluid intelligence remain statistically significant, which could be due to the reduced sample size.

In previous studies among adults older than 65 years old [5, 20, 21], it’s hypothesized that DNAm age acceleration influences cognitive functions through numerous mechanisms such as synaptic plasticity and neurogenesis and immune dysfunction [31, 32]. Our study showed that crystallized intelligence measured by PPVT-15 is associated with age acceleration measured 35 years later, demonstrating the possibility of a reverse causation or even a bidirectional relationship, that is, the possibility that cognitive function at a younger age could be a cause rather than just the consequences of biological aging. Further research with more time points of cognitive function and DNAm age acceleration is needed to establish causality.

The effect of early crystallized intelligence on later biological aging could be through its influence in daily stress processes, behavior, lifestyle or health [33], which might be starting to accumulate during adolescence. For example, a higher crystallized cognition may increase the likelihood of exposure to daily stressors [33], which could influence DNAm age acceleration [34]. The attenuated association between adolescent crystallized intelligence and GrimAge acceleration after adjusting for adult SES suggests that adult SES could be a potential mediator (Supplementary Figure 6A) and improving SES could be helpful for slowing down some aspects of biological aging associated with cognition at younger ages. However, the attenuated association could also be due to unmeasured confounders in our study that induce confounding or collider bias through adult SES (Supplementary Figure 6B, 6C).

On the other hand, accelerated aging is associated with both crystallized and fluid intelligence in midlife. Crystallized intelligence measured by WTAR in midlife is statistically associated with PhenoAge and DunedinPACE but not GrimAge. This observed heterogeneity may be explained by the inherent difference in the methodology used to construct the epigenetic clocks. Early cognition may be more related to biological aging in response to physiological risk factors and stress factors [9], while later cognition may be more related to biological aging in terms of phenotypic changes and organ failures [8, 35]. GrimAge, PhenoAge and DunedinPACE are significantly associated with fluid intelligence measured by DS, and all associations are robust to sensitivity analyses. This suggests that it is possible that fluid intelligence, which refers to the capacity to process complex information involved in reasoning and problem-solving tasks, may be more affected by accelerated aging than language processing skills. Midlife DNAm age acceleration may influence midlife crystallized and fluid intelligence through inflammatory or oxidative stress pathways [36].

Previous studies in older population found that faster decline of memory and executive function are associated with Horvath and Hannum DNAm age acceleration [5, 20], but neither of these measures are predictive of any cognitive function measure in our study. This could be due to a smaller sample size or usage of different cognition measures in our study. Conversely, our results suggest that a more progressive DNAm age acceleration measure that indicates mortality or morbidity such as GrimAge, PhenoAge and DunedinPACE may be more sensitive to changes in cognitive function compared to DNAm age acceleration that only considers chronological age.

To the best of our knowledge, this is the first study to show the association of cognition at younger age with midlife age acceleration, and associations between midlife age acceleration measures and cognitive function that are independent of childhood and adolescent cognition. This aligns with our hypothesis that cognitive function at early age may affect aging in midlife and in turn affect cognitive function in midlife. However, we only have one time point of DNAm in midlife and no information on cognition, DNAm or other covariates during the 35-year gap. This limits the inference on the causal relationship between cognitive function and DNAm age acceleration. Selection of participants into the DISPAR study at midlife was limited to those who continued to live in California at midlife, which may have introduced selection bias [3]. Another limitation that warrants mention is that the fluid intelligence was only administered in childhood, so we could not assess its association with DNAm age acceleration in midlife. Additionally, the measures of crystalized and fluid intelligence were different between childhood/adolescence and midlife, so we were not able to efficiently describe cognitive function trajectories over time. The differences in measures may also account for the observed difference in their associations with DNAm age acceleration.

In conclusion, our study brings attention to the potential influence of adolescent crystalized intelligence on age-related DNAm at older age. We showed a potential relationship of both crystalized and fluid intelligence with aging acceleration in midlife. Moreover, we demonstrated that GrimAge, PhenoAge, DunedinPACE age acceleration measures could prove valuable for studying the relationship between aging and cognitive function. Future studies with longitudinal cognitive functions and DNAm age accelerations at more time points across the lifespan are needed to decipher their relationships.

## MATERIALS AND METHODS

### Study population

Our study utilized data from adult offspring of mothers that were recruited in the Child Health and Development Studies (CHDS), which was a prospective study of pregnant women seeking prenatal care from the Kaiser Foundation Health Plan in Oakland, California from 1959 to 1966 [37]. This original cohort was comprised of 19,044 live births from participating mothers with diverse race/ethnic background. Extensive sociodemographic, behavioral and clinical information on the offspring and their mothers were collected through in-person interviews and medical records [37]. Follow-up assessments of cognitive function in specific subsets of offspring were conducted subsequently at age 5, 9–11, and 15–17 years.

After approximately 35 years, a subset of the adult offspring who had assessments during adolescence were selected for another follow-up examination and interview for the CHDS Disparities (DISPAR) study [38]. Among 985 eligible pool that could be located and recruited by phone, 605 were successfully enrolled and participated in a telephone interview, 510 completed a home visit and 497 completed a self-administered questionnaire. At the home visit, participants completed a short cognitive battery and a blood draw that resulted in 400 viable serology samples after excluding participants who refused or had a technical issue during the draw [39].

The current study, DISPAR Aging, builds from the CHDS DISPAR study by using the blood samples it collected to profile for DNA methylation. From the sample of 400, we further excluded adult offspring who refused DNA analysis or use of their blood samples for future studies, resulting in a final analytic sample of 359.

### Cognitive function measures

Childhood cognitive function was measured by two tests, the Raven Colored Progressive Matrices test at the age of 9–11 years (RCPM-9) and the Peabody Picture Vocabulary test at the age of 9–11 years (PPVT-9) [3]. RCPM-9 consisted of series of items each containing a matrix display of 12 graphical patterns where one of them was missing. The children were provided with 6 alternative patterns and asked to choose the best that fits the missing element. It is a test for logical and perceptual reasoning and is typically used to assess fluid intelligence [40]. In PPVT-9 test, participants were shown a series of sheets with 4 pictures and were asked to indicate which best represents a target word said by the examiner. This is a test for vocabulary and language processing, which is typically used to assess crystallized intelligence [41]. Adolescent cognitive function was only measured by the Peabody Picture Vocabulary Test at the age of 15–17 years (PPVT-15) [3]. The RCPM-9 raw score can range from 0–60. The average range for PPVT raw scores is 72.28–92.18 at the age of 11 years and 90.98–119.24 at the age of 17 years.

Three cognitive function measures were assessed in midlife, including the Wechsler Test of Adult Reading (WTAR) [27], Verbal Fluency (VF) test [28], the Wechsler Adult Intelligence Scale-Revised Digit Symbol (DS) test [29]. WTAR intended to measure participants’ intellectual functioning, reading and recognition abilities by asking the participants to pronounce a list of 50 phonetically incorrect words [27]. VF was typically used to evaluate participants’ executive functions, semantic fluency, and speed of processing by asking participants to name as many animals as possible within 1 minute [28]. For DS test, each participant was presented with a sheet with a number-to-symbol key at the top, along with a grid of numbers and blank boxes, and was asked to write down as many correct corresponding symbols underneath each number. This test measures participants’ executive functions and speed of processing [29]. The WTAR and DS test can range from 0–50 and 0–133 respectively. There is no limit for the maximum VF test score, however, a VF test score less than 15 could indicate potential cognitive impairment [42]. Details of the cognitive measures have been previously published [3]. Spearman correlations were calculated between cognitive function measures.

### DNAm age acceleration in midlife

Genomic DNA was extracted from whole blood samples using with the PureLink^®^ Genomic DNA Mini Kit by Invitrogen (Carlsbad, CA, USA). DNA methylation was measured using 850K BeadChips following the standard protocol. Intensity files in IDAT format were imported in R using minfi package [43]. Intensity values with a detection *P*-value ≥ 0.001 were set to missing for each DNAm site and DNAm sites missing in more than 5% of the samples were removed. All samples passed a call rate of 95%. Normal-exponential out-of-band background correction was used to account for technical variation in the intensity values, which were then used to generate a *β* value for each DNAm site. We calculated the proportions of 6 cell types including CD4+ T cells, CD8+ T cells, natural killer T cells, B cells, monocytes, and granulocytes in blood by applying algorithm developed by Houseman et al to the DNAm data [44].

We considered 5 different measures of DNAm age acceleration, including Horvath [10], Hannum [7], GrimAge [9], PhenoAge [8] and DunedinPACE [35]. For Horvath [10] and Hannum [7] epigenetic clocks, the difference in DNAm between younger and older people represents biological processes of aging while GrimAge and PhenoAge epigenetic clocks are using the difference in DNAm patterns that predict mortality and health, respectively, to represent biological processes of aging [8, 9]. These four epigenetic clocks were generated by uploading the DNAm *β* values of 30,084 prespecified DNAm sites and information on age and sex to the online DNA Methylation Age Calculator (https://dnamage.genetics.ucla.edu/) with the “normalization” and “advanced analysis in blood” options. DNAm age accelerations were calculated as the residuals in epigenetic clocks after regressing out chronological age, thus a positive DNAm age acceleration reflects more rapid biological aging.

Recently, Belsky et al. applied elastic-net regression to develop a DNA methylation model that predicts the pace of aging, which was quantified from 19 organ-system integrity data of four time points spanning two decades [35]. This resulted in a new epigenetic biomarker named DunedinPACE, with values larger than 1 indicating faster aging and values less than 1 indicating slower aging. The idea behind DunedinPACE is that the DNAm responses to the variation in function decline of multiple organ systems represents the biological processes of aging. DunedinPACE measures someone’s pace of aging in 20 years while the other clock-based age acceleration measures the accumulated years of aging at the time of assessment in a cross-sectional setting. We calculated DunedinPACE using the DunedinPACE R package. Spearman correlations were calculated between DNAm age acceleration measures.

### Additional covariates

#### Childhood and adolescence

Participants’ exact age, school grade level and BMI at the time of cognition assessment were recorded. A composite score for childhood/adolescence socioeconomic status (SES) was created based on maternal education at birth, paternal occupation, and family income at birth, ages 9–11 and 15–17 [38]. At the age of 15–17 years, participants were asked how often they had consumed alcoholic beverage in the past 6 months and those who answered at least one drink were defined as current drinkers. They were also asked if they had ever ‘regularly’ smoked at least 1 cigarette daily and if they currently smoked cigarettes. Alcohol and cigarette use at age 9–11 was not recorded. Additionally, during interviews in midlife, participants were asked to recall if they had lived with a regular cigarette, cigar, or pipe smoker who smoked in their home when they were young. We note that information on BMI, alcohol use and personal cigarette use in childhood and adolescence was only used in multiple imputation but not in analyses examining cognition and age acceleration associations (see multiple imputation and statistical analyses method section for more details).

#### Midlife

Participants’ exact age and BMI were collected at the time of midlife cognition assessment. Midlife SES index was developed by combining information on college education, self-reported family income and occupational status. Details on how SES indices were developed have been described in detail in a prior publication [39]. Participants were asked about alcohol and cigarette use during the midlife interviews in DISPAR. If they had ever consumed any alcoholic beverages at least once a month for six months over the past 12 months, they were defined as current drinkers. If they had done so but not over the past 12 months, they were defined as past drinkers. Otherwise, they were categorized as “never drink”. Participants were also asked if they had ever smoked at least 1 cigarette per day for one month or more and if they smoked at any time in the past 12 months. If they answered yes to both questions, they were defined as current smokers. If they answered yes to the first question but no to the second question, they were defined as past smokers. Otherwise, they were categorized as “never smokers”. Blood pressure was measured during the home visit and hypertension was defined as having systolic blood pressure over or equal to 120 mm Hg or diastolic blood pressure over or equal to 80 mm Hg, or currently taking hypertension medication.

### Multiple imputation

Of the 359 participants who had DNAm data, 26 were missing RCPM-9, 34 were missing PPVT-9, 61 were missing PPVT-15 and 19 were missing WTAR. We found that RCPM-9, PPVT-9, PPVT-15 and WTAR were positively correlated (Supplementary Figure 2A), thus, we used multiple imputation by chained equations to impute missing values on cognitive function and other covariates. Multiple imputation was performed among participants that were successfully enrolled in DISPAR, with or without DNAm data, and had at least one measure of RCPM-9, PPVT-9, PPVT-15 or WTAR (*n* = 599, Figure 1). We assumed missing at random and performed multiple imputation using Predictive mean matching by MICE package in R [45]. We assumed variables at the same time point can predict each other and variables from different time points cannot predict each other except for the variables that measure related information, such as adolescent and midlife smoking status. The predictor matrix is presented in Supplementary Table 1. Twenty complete datasets were imputed, and estimates obtained by statistical analyses were combined using “pool” function in the MICE package in R [45]. The convergence of cognitive function measures is shown in Supplementary Figure 7.

### Statistical analyses

All analyses were conducted in R (4.3.0). Potential confounders were identified through literature review and directed acyclic graphs (DAG) (Supplementary Figure 8A–8C).

#### Childhood and adolescence

Linear regressions were used to assess associations of each childhood or adolescent cognitive function measure (independent variable) with each DNAm age acceleration measure in midlife (dependent variable). All models were adjusted for age and school grade level at assessment, sex, race, household smoking status and cell type proportions. According to the DAG for adolescent cognitive function, BMI, alcohol use and personal cigarette use in adolescence could be potential mediators [46–49] (Supplementary Figure 8B). Thus, these covariates were not included in the linear regressions but were used in multiple imputation. In a separate model, we additionally adjusted for adult SES to assess its impact on the associations between adolescent cognition and midlife aging. In order to adjust for multiple testing, Benjamini-Hochberg false discovery rate (FDR) adjustment was applied and FDR-*q*-values < 0.05 were used to define statistical significance. Sensitivity analyses by complete case analyses (*n* = 286) were performed to test the robustness of the associations.

#### Midlife

Another set of linear regressions were used to assess associations of each DNAm age acceleration measure in midlife with each midlife cognitive function measure. Similar to the majority of the current literature, we hypothesize that age acceleration in midlife reflects general deterioration and has an impact on cognitive function in midlife. Thus, in these analyses, age acceleration was treated as the independent variable and cognitive function was treated as the dependent variable. All models were adjusted for age at blood draw, sex, race, SES, BMI, alcohol and cigarette use, RCPM-9, PPVT-9, PPVT-15 and cell type proportions. We included RCPM-9, PPVT-9 and PPVT-15 in the models because we wanted to evaluate the associations in midlife independent of the influence from cognition at younger age. Unlike in childhood and adolescence, BMI, alcohol, and cigarette use in midlife are likely to be confounders (Supplementary Figure 8C), and thus were adjusted in the models. Sensitivity analyses by complete case analyses (*n* = 211) were performed to test the robustness of the associations.

## Supplementary Materials

Supplementary Figures

Supplementary Table 1
