# Supplementary material for: Associations of childhood, adolescence, and midlife cognitive function with DNA methylation age acceleration in midlife
Source: Aging (Albany NY). 2024 Jun 13;16(11):9350–68. doi: 10.18632/aging.205943 (PMC11210249; doi:10.18632/aging.205943)
Supplement: Supplementary Figures [file aging-16-205943-s001.pdf]

SUPPLEMENTARY FIGURES

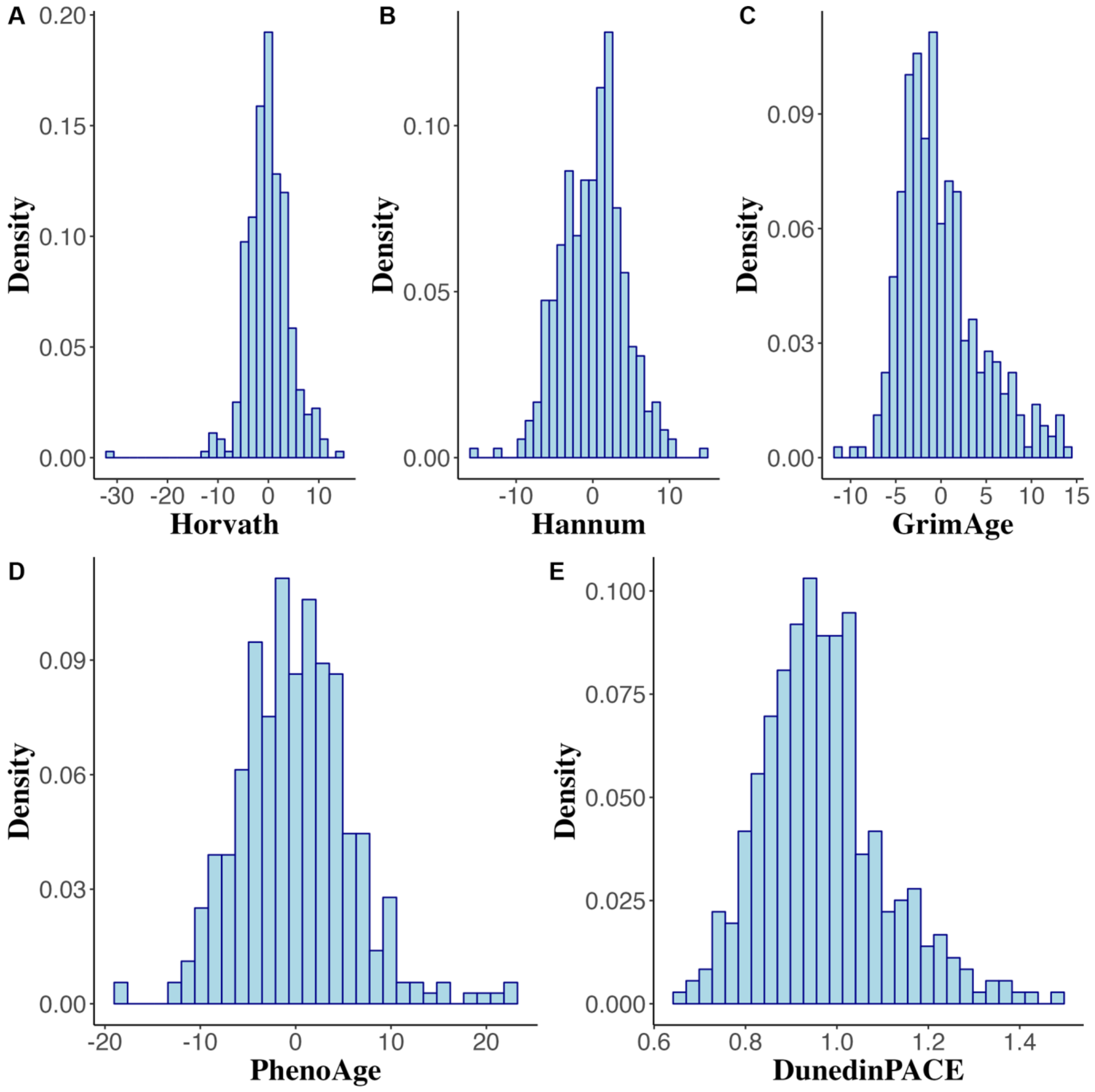

Supplementary Figure 1. Distribution of DNA methylation age acceleration measures in midlife, including (**A**) Horvath, (**B**) Hannum, (**C**) GrimAge, (**D**) PhenoAge, (**E**) DunedinPACE.

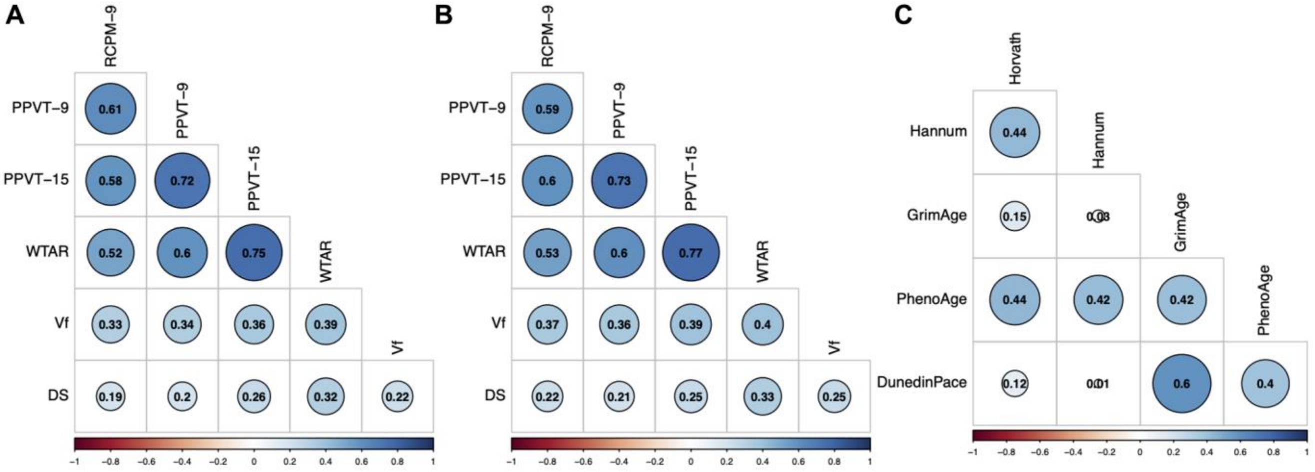

Supplementary Figure 2. Heatmaps showing correlations between adolescent cognitive function measures (**A**) before (participants without missing values in any cognitive function measure, *n* = 269) and (**B**) after multiple imputation (*n* = 359), and (**C**) between midlife DNA methylation age acceleration measures (*n* = 359).

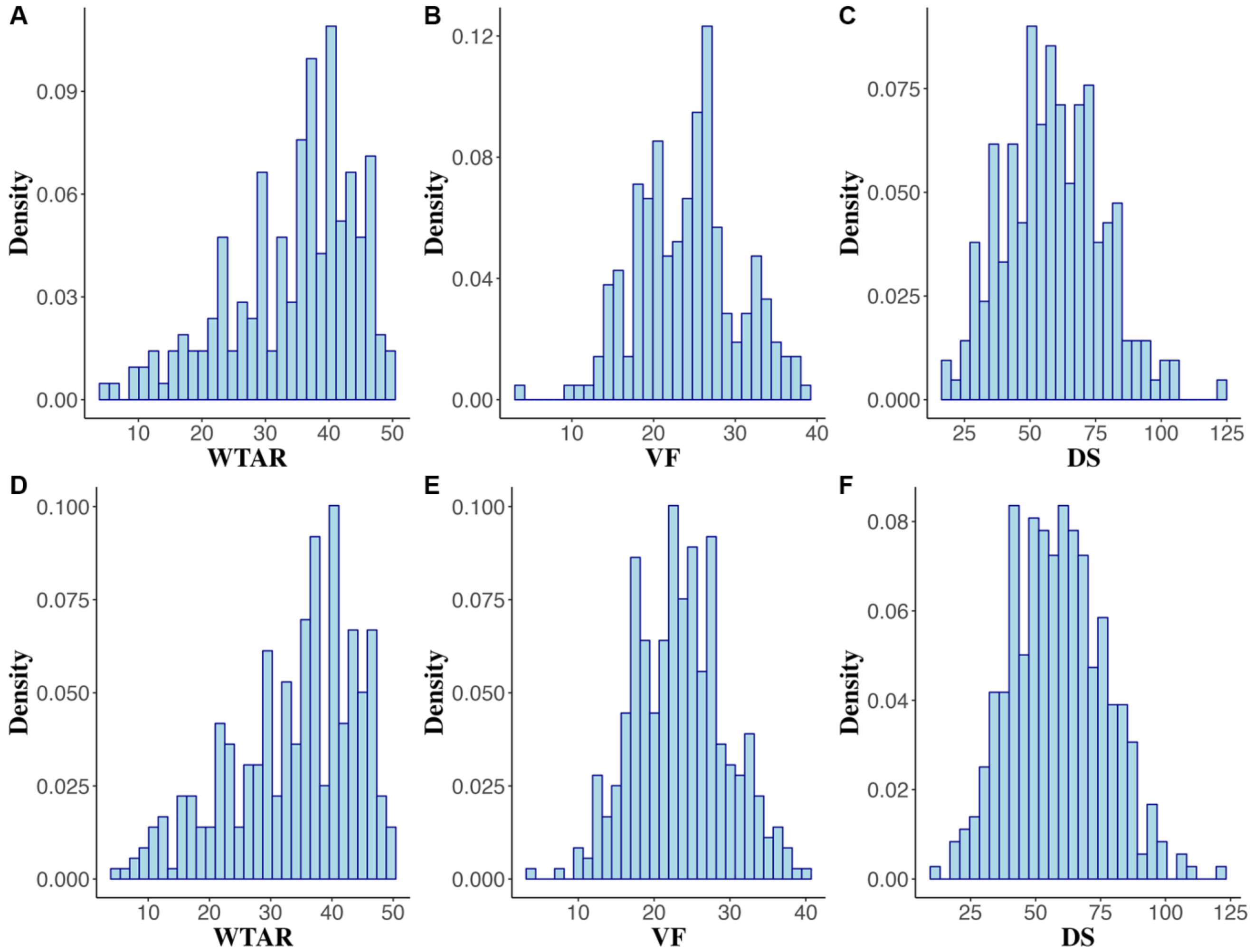

Supplementary Figure 3. Distributions of cognitive function in midlife. These include (**A**) Wechsler Test of Adult Reading (WTAR), (**B**) Verbal Fluency (VF), (**C**) Digit Symbol (DS) before multiple imputation (*n* = 211) and (**D**) WTAR, (**E**) VF, (**F**) DS after multiple imputation (*n* = 359).

Cognition	Age acceleration	Beta (95% CI)	Change	P-value	FDR-q
RCPM-9	Horvath	-0.031 (-0.086 to 0.024)	-0.72% SD	0.27	0.63
RCPM-9	Hannum	0.032 (-0.012 to 0.075)	0.77% SD	0.16	0.54
RCPM-9	GrimAge	-0.045 (-0.095 to 0.005)	-1.03% SD	0.08	0.54
RCPM-9	PhenoAge	-0.024 (-0.096 to 0.048)	-0.41% SD	0.51	0.85
RCPM-9	DunedinPACE	-0.008 (-0.023 to 0.007)	-0.6% SD	0.29	0.63
PPVT-9	Horvath	-0.000 (-0.052 to 0.051)	-0.01% SD	0.99	0.99
PPVT-9	Hannum	-0.006 (-0.046 to 0.035)	-0.13% SD	0.79	0.95
PPVT-9	GrimAge	-0.006 (-0.053 to 0.041)	-0.14% SD	0.80	0.95
PPVT-9	PhenoAge	0.008 (-0.060 to 0.075)	0.13% SD	0.82	0.95
PPVT-9	DunedinPACE	-0.010 (-0.024 to 0.004)	-0.71% SD	0.18	0.54
PPVT-15	Horvath	-0.014 (-0.051 to 0.024)	-0.32% SD	0.47	0.85
PPVT-15	Hannum	0.008 (-0.022 to 0.037)	0.18% SD	0.62	0.93
PPVT-15	GrimAge	-0.044 (-0.077 to -0.010)	-1% SD	0.01	0.16
PPVT-15	PhenoAge	0.002 (-0.046 to 0.051)	0.04% SD	0.92	0.99
PPVT-15	DunedinPACE	-0.007 (-0.017 to 0.003)	-0.52% SD	0.17	0.54

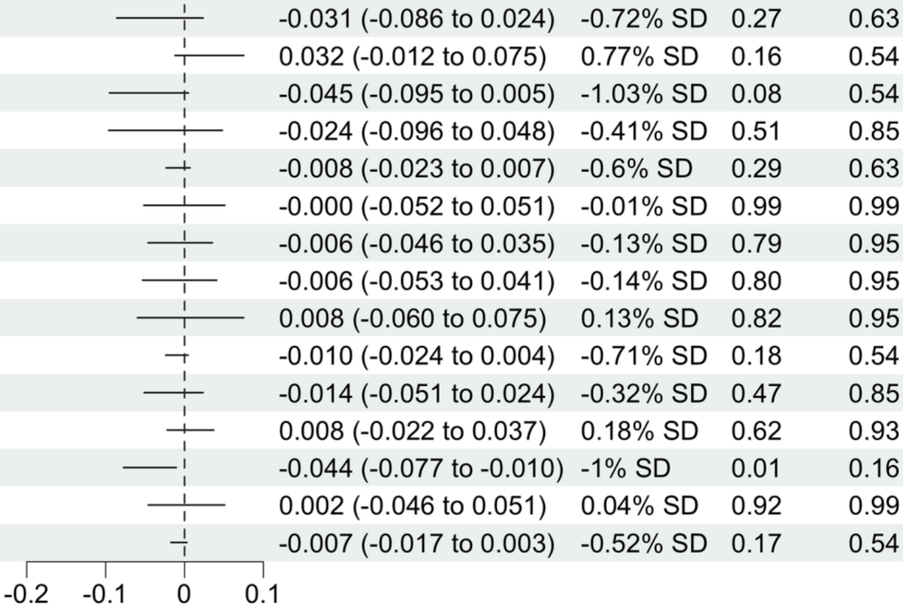

Supplementary Figure 4. Sensitivity analyses of the associations between childhood/adolescent cognitive function and midlife DNA methylation age accelerations by complete case analysis (*n* = 286). Betas multiplied by 10 were shown for associations with DunedinPACE for better visualization. Column "Change" is showing that 1-unit change in childhood or adolescent cognitive function is associated with X.XX% standard deviation (SD) change in DNA methylation age acceleration. SD for each DNA methylation age can be found in Table 1.

Cognition	Age acceleration	Beta (95% CI)	Change	P-value	FDR-q
WTAR	Horvath	-0.154 (-0.372 to 0.064)	-0.67% SD	0.17	0.37
WTAR	Hannum	-0.171 (-0.421 to 0.078)	-0.7% SD	0.18	0.37
WTAR	GrimAge	-0.142 (-0.387 to 0.104)	-0.62% SD	0.26	0.43
WTAR	PhenoAge	-0.103 (-0.258 to 0.052)	-0.61% SD	0.2	0.37
WTAR	DunedinPACE	-0.061 (-0.140 to 0.019)	-0.83% SD	0.14	0.37
VF	Horvath	0.024 (-0.184 to 0.233)	0.1% SD	0.82	0.82
VF	Hannum	-0.028 (-0.267 to 0.211)	-0.11% SD	0.82	0.82
VF	GrimAge	-0.054 (-0.289 to 0.181)	-0.24% SD	0.65	0.75
VF	PhenoAge	-0.066 (-0.214 to 0.083)	-0.39% SD	0.39	0.53
VF	DunedinPACE	-0.036 (-0.112 to 0.040)	-0.5% SD	0.35	0.52
DS	Horvath	-0.234 (-0.871 to 0.403)	-1.02% SD	0.47	0.59
DS	Hannum	-0.999 (-1.714 to -0.283)	-4.1% SD	0.01	0.03
DS	GrimAge	-1.121 (-1.821 to -0.421)	-4.92% SD	2e-03	0.01
DS	PhenoAge	-0.662 (-1.106 to -0.218)	-3.93% SD	4e-03	0.02
DS	DunedinPACE	-0.363 (-0.590 to -0.136)	-4.94% SD	2e-03	0.01

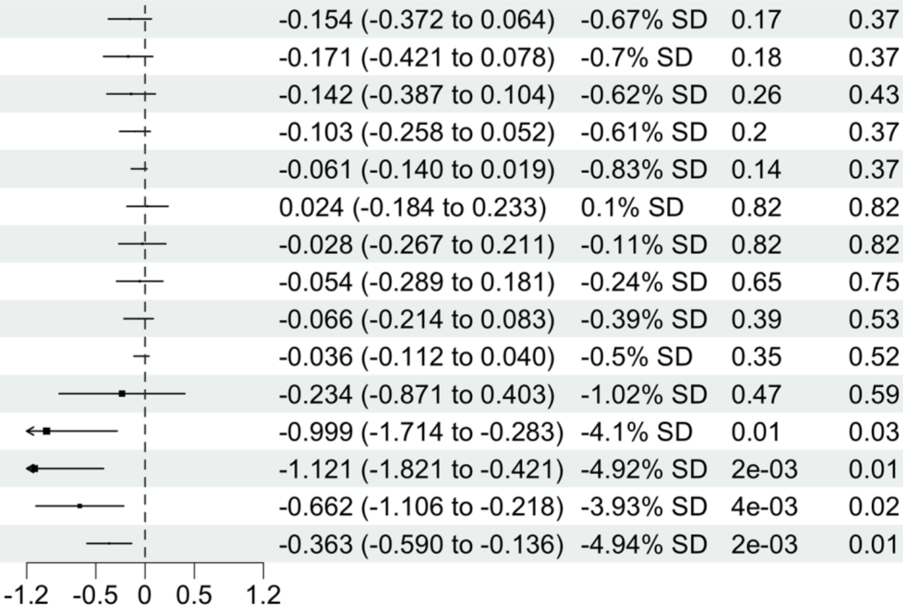

Supplementary Figure 5. Sensitivity analyses of the associations between midlife cognitive function and midlife DNA methylation age accelerations by complete case analysis (*n* = 211). Betas divided by 100 were shown for associations with DunedinPACE for better visualization. Column "Change" is showing that 1-standard deviation (SD) change in DNA methylation age acceleration is associated with X.XX-unit change in midlife cognitive function. SD for each DNA methylation age can be found in Table 1.

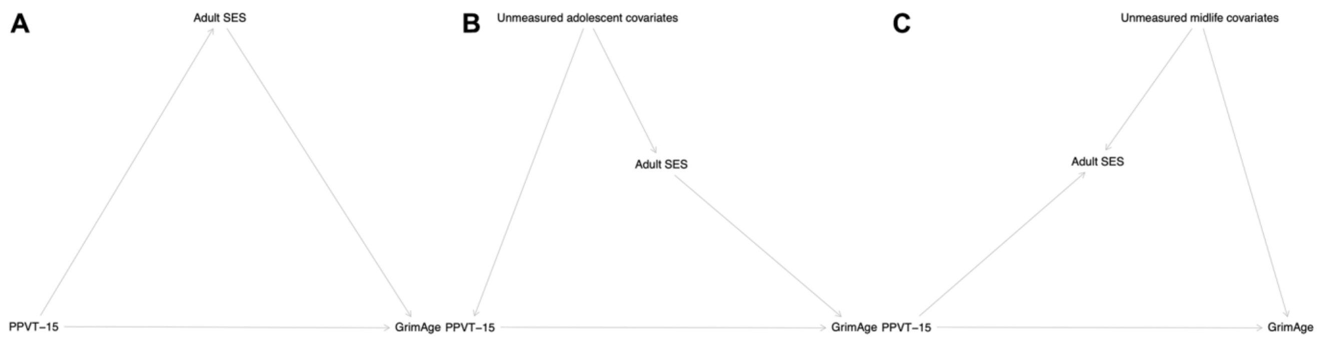

Supplementary Figure 6. Directed acyclic graphs (DAGs) for possible causal relationship between adolescent cognitive function, Adult SES and midlife DNA methylation age acceleration. (A) SES is a mediator. (B) SES is associated with confounders. (C) SES is a collider.

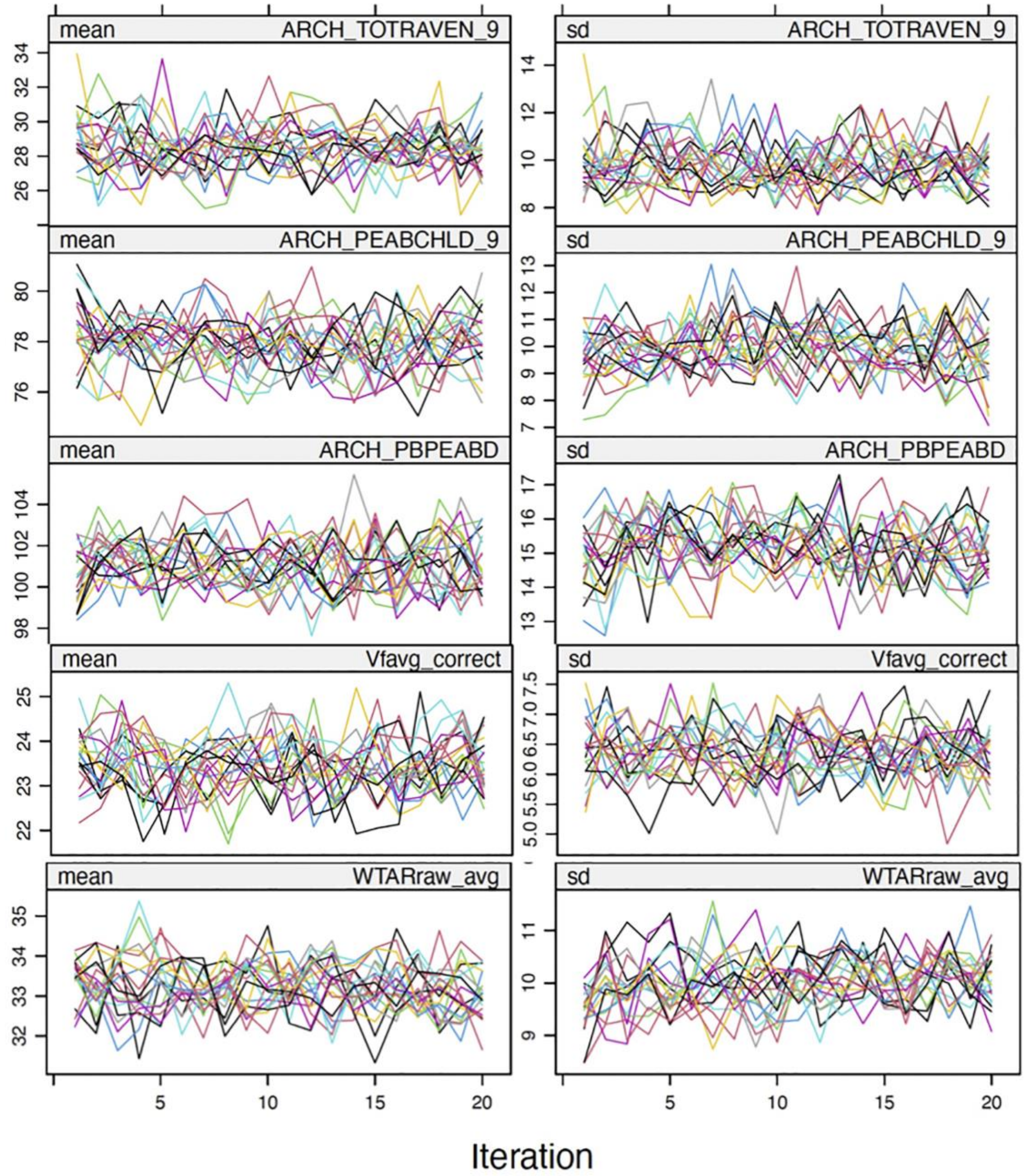

Supplementary Figure 7. Traces of convergence of cognitive function measures in multiple imputation algorithm.

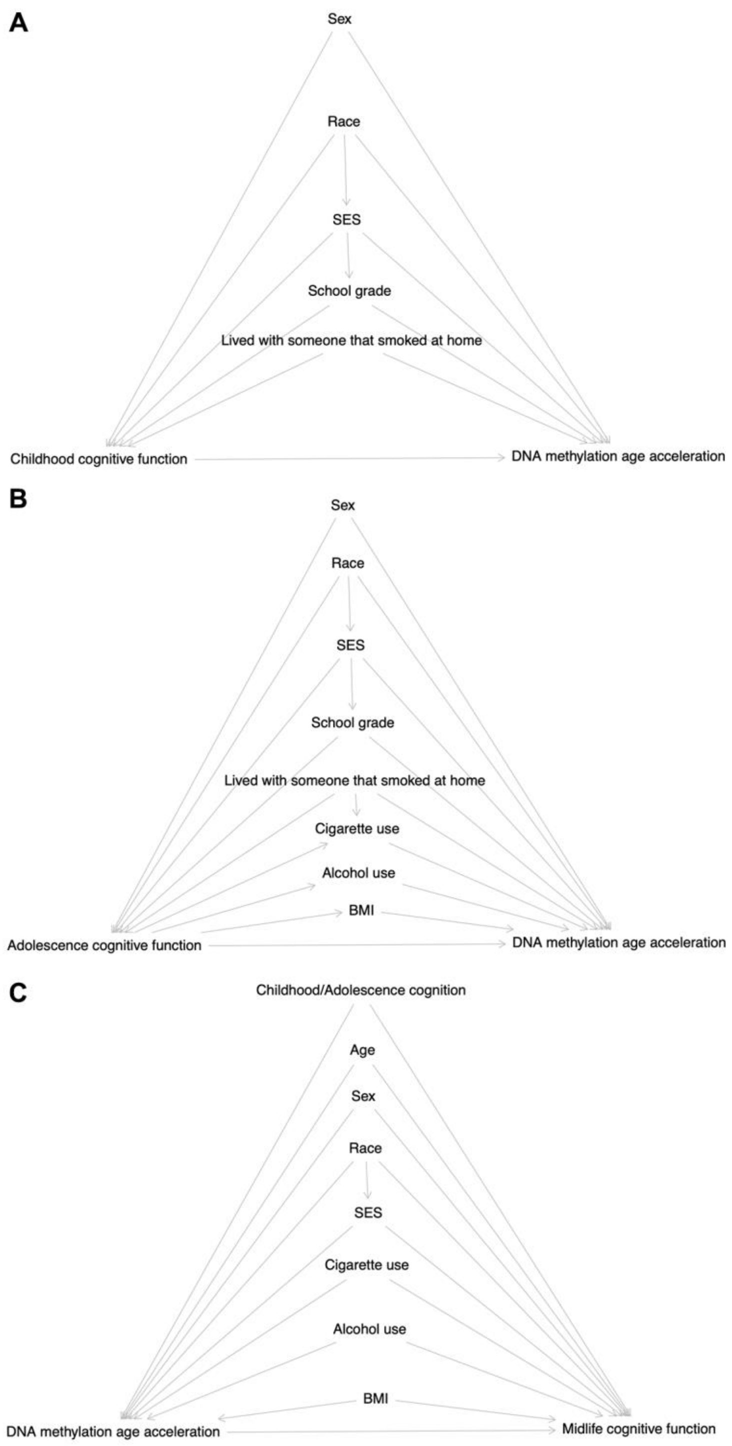

Supplementary Figure 8. Directed acyclic graphs (DAGs) conceptualizing the causal relationships between (**A**) childhood, (**B**) adolescence, (**C**) midlife cognitive function and midlife DNA methylation age accelerations.
