# Supplementary material for: Associations of childhood, adolescence, and midlife cognitive function with DNA methylation age acceleration in midlife
Source: Aging (Albany NY). 2024 Jun 13;16(11):9350–68. doi: 10.18632/aging.205943 (PMC11210249; doi:10.18632/aging.205943)
Supplement: Supplementary Table 1 [file aging-16-205943-s002.docx]

Supplementary Table 1. Predictor matrix for multivariate imputation by chained equations. “X” specifies the columns used as predictors for imputation of the targeted rows.

|  | Sex | Race | Childhood age | Childhood grade | Adolescence age | Childhood Adolescence SES | Adolescence grade |
| --- | --- | --- | --- | --- | --- | --- | --- |
| Sex |  |  |  |  |  |  |  |
| Race | X |  | X | X | X | X | X |
| Childhood age |  |  |  |  |  |  |  |
| Childhood grade | X | X | X |  |  | X |  |
| Adolescence age |  |  |  |  |  |  |  |
| Childhood/Adolescence SES |  |  |  |  |  |  |  |
| Adolescence grade | X | X |  |  | X | X |  |
| Adolescence alcohol use | X | X |  |  | X | X | X |
| Lived with someone that smoke at home (Childhood, Adolescence) |  | X |  |  |  | X |  |
| Adolescence cigarette use | X | X |  |  | X | X | X |
| Childhood BMI | X | X | X |  |  | X |  |
| Adolescence BMI | X | X |  |  | X | X |  |
| RCPM-9 | X | X | X | X |  | X |  |
| PPVT-9 | X | X | X | X |  | X |  |
| PPVT-15 | X | X |  |  | X | X | X |
| Midlife age |  |  |  |  |  |  |  |
| Midlife SES |  |  |  |  |  |  |  |
| Midlife BMI | X | X |  |  |  |  |  |
| Ever smoked at least 1 cigarette per day for one month or more? | X | X |  |  |  |  |  |
| Smoke in the past 12 months? | X | X |  |  |  |  |  |
| Midlife alcohol use | X | X |  |  |  |  |  |
| Midlife hypertension | X | X |  |  |  |  |  |
| WTAR | X | X |  |  |  |  |  |
| VF | X | X |  |  |  |  |  |

|  | Adolescence alcohol use | Lived with someone that smoke at home (Childhood, Adolescence) | Adolescence cigarette use | Childhood BMI | Adolescence BMI | RCPM-9 |
| --- | --- | --- | --- | --- | --- | --- |
| Sex |  |  |  |  |  |  |
| Race | X | X | X | X | X | X |
| Childhood age |  |  |  |  |  |  |
| Childhood grade |  |  |  |  |  | X |
| Adolescence age |  |  |  |  |  |  |
| Childhood/Adolescence SES |  |  |  |  |  |  |
| Adolescence grade |  |  |  |  |  | X |
| Adolescence alcohol use |  | X | X | X | X | X |
| Lived with someone that smoke at home (Childhood, Adolescence) | X |  | X | X | X | X |
| Adolescence cigarette use | X | X |  | X | X | X |
| Childhood BMI | X | X | X |  | X | X |
| Adolescence BMI | X | X | X | X |  | X |
| RCPM-9 | X | X | X | X |  |  |
| PPVT-9 | X | X | X | X |  | X |
| PPVT-15 | X | X | X |  | X | X |
| Midlife age |  |  |  |  |  |  |
| Midlife SES |  |  |  |  |  |  |
| Midlife BMI |  |  |  | X | X |  |
| Ever smoked at least 1 cigarette per day for one month or more? |  | X | X |  |  |  |
| Smoke in the past 12 months? |  | X | X |  |  |  |
| Midlife alcohol use | X |  |  |  |  |  |
| Midlife hypertension |  |  |  |  |  |  |
| WTAR |  |  |  |  |  | X |
| VF |  |  |  |  |  | X |

|  | PPVT-9 | PPVT-15 | Midlife age | Midlife SES | Midlife BMI | Ever smoked at least 1 cigarette per day for one month or more? | Smoke in the past 12 months? |
| --- | --- | --- | --- | --- | --- | --- | --- |
| Sex |  |  |  |  |  |  |  |
| Race | X | X | X | X | X | X | X |
| Childhood age |  |  |  |  |  |  |  |
| Childhood grade | X |  |  |  |  |  |  |
| Adolescence age |  |  |  |  |  |  |  |
| Childhood/Adolescence SES |  |  |  |  |  |  |  |
| Adolescence grade | X | X |  |  |  |  |  |
| Adolescence alcohol use | X | X |  |  |  |  |  |
| Lived with someone that smoke at home (Childhood, Adolescence) | X | X |  |  |  | X | X |
| Adolescence cigarette use | X | X |  |  |  | X | X |
| Childhood BMI | X | X |  |  | X |  |  |
| Adolescence BMI | X | X |  |  | X |  |  |
| RCPM-9 | X | X |  |  |  |  |  |
| PPVT-9 |  | X |  |  |  |  |  |
| PPVT-15 | X |  |  |  |  |  |  |
| Midlife age |  |  |  |  |  |  |  |
| Midlife SES |  |  |  |  |  |  |  |
| Midlife BMI |  |  | X | X |  | X | X |
| Ever smoked at least 1 cigarette per day for one month or more? |  |  | X | X | X |  | X |
| Smoke in the past 12 months? |  |  | X | X | X | X |  |
| Midlife alcohol use |  |  | X | X | X | X | X |
| Midlife hypertension |  |  | X | X | X | X | X |
| WTAR | X | X | X | X | X | X | X |
| VF | X | X | X | X | X | X | X |

|  | Midlife alcohol use | Midlife hypertension | WTAR | VF |
| --- | --- | --- | --- | --- |
| Sex |  |  |  |  |
| Race | X | X | X | X |
| Childhood age |  |  |  |  |
| Childhood grade |  |  |  |  |
| Adolescence age |  |  |  |  |
| Childhood/Adolescence SES |  |  |  |  |
| Adolescence grade |  |  |  |  |
| Adolescence alcohol use | X |  |  |  |
| Lived with someone that smoke at home (Childhood, Adolescence) |  |  |  |  |
| Adolescence cigarette use |  |  |  |  |
| Childhood BMI |  |  |  |  |
| Adolescence BMI |  |  |  |  |
| RCPM-9 |  |  | X | X |
| PPVT-9 |  |  | X | X |
| PPVT-15 |  |  | X | X |
| Midlife age |  |  |  |  |
| Midlife SES |  |  |  |  |
| Midlife BMI | X | X | X | X |
| Ever smoked at least 1 cigarette per day for one month or more? | X | X | X | X |
| Smoke in the past 12 months? | X | X | X | X |
| Midlife alcohol use |  | X | X | X |
| Midlife hypertension | X |  | X | X |
| WTAR | X | X |  | X |
| VF | X | X | X |  |
